# A Wireless Sensor Network Based Personnel Positioning Scheme in Coal Mines with Blind Areas

**DOI:** 10.3390/s101109891

**Published:** 2010-11-03

**Authors:** Zhigao Liu, Chunwen Li, Danchen Wu, Wenhan Dai, Shaobo Geng, Qingqing Ding

**Affiliations:** 1 Department of Automation, Tsinghua University, Beijing 100084, China; E-Mail: lcw@mail.tsinghua.edu.cn; 2 Department of Electrical Engineering, Tsinghua University, Beijing 100084, China; E-Mails: wdc04@mails.tsinghua.edu.cn (D.C.W.); shaobo813@126.com (S.B.G.); d-qq00@mails.tsinghua.edu.cn (Q.Q.D.); 3 Department of Electronic Engineering, Tsinghua University, Beijing 100084, China; E-Mail: daiwenhan212@hotmail.com

**Keywords:** personnel global positioning, wireless sensor networks, tunnel network model, global positioning method

## Abstract

This paper proposes a novel personnel positioning scheme for a tunnel network with blind areas, which compared with most existing schemes offers both low-cost and high-precision. Based on the data models of tunnel networks, measurement networks and mobile miners, the global positioning method is divided into four steps: (1) calculate the real time personnel location in local areas using a location engine, and send it to the upper computer through the gateway; (2) correct any localization errors resulting from the underground tunnel environmental interference; (3) determine the global three-dimensional position by coordinate transformation; (4) estimate the personnel locations in the blind areas. A prototype system constructed to verify the positioning performance shows that the proposed positioning system has good reliability, scalability, and positioning performance. In particular, the static localization error of the positioning system is less than 2.4 m in the underground tunnel environment and the moving estimation error is below 4.5 m in the corridor environment. The system was operated continuously over three months without any failures.

## 1. Introduction

Coal mine operations have been recognized as one of the most dangerous working environments due to the poor ventilation, potential rock falls, and presence of toxic gases. In this regard, the real-time localization of miners can greatly improve the daily management level and rescue efficiency in emergency situations, and has been applied in more and more underground mines.

As a part of a mine monitoring system, a coal mine personnel positioning system based on the wireless technology [1] (mostly the Radio Frequency Identification—RFID—technology), is implemented by reading and registering miners' RFID card information with a number of readers situated within the key regions of the mine, and the locations of the readers are taken as the miners’ location [2,3]. Such positioning systems cannot achieve high positioning accuracy as they can only tell whether the miners are between two readers or not. Although increasing the number of the readers along the pathway may help improve the accuracy, the cost associated with the deployment, management and maintenance costs of such a system will be very high (e.g., over 75% of the installation time will be spent on the system wires and cables [4]).

Wireless sensor networks (WSN) [5] have been drawing increasing attention due to many successful applications [6,7], and the integration of WSN with mine personnel positioning has been widely investigated to overcome the above limitations. Tian *et al.* proposed to model the coal mine as a one-dimensional vector and the personnel location was represented by the distance between the person and the tunnel endpoint [8]. Wireless beacon nodes were evenly deployed in the mine tunnel, and kept sending hello messages to the mobile node, which calculated its own position according to the received messages. The positioning method based on TOA (Time of Arrival: TOA) was proposed by Li *et al.* [9]. The distance between the blind node and the beacon nodes was calculated by multiplying the measured transmission time by the electromagnetic wave propagation speed (3 × 10^8^ m/s). In [10], a fingerprint method was applied in the underground mine, where off-line training data were used to create a reference database of possible locations. The localization can be done by choosing the reference point from the database that matches best with the observed data. Moreover, the ultra wide band (Ultra Wide Band: UWB)-based WSN for localization in mining environments was presented by Abdellah *et al.* [11]. The received signal strength indication (Received Signal Strength Indication: RSSI)-based location algorithm was optimized by Pei *et al.* [12], Yang *et al.* [13] and Zhang *et al.* [14] to improve positioning accuracy.

For practical applications, several issues exist in current designs and deployment schemes with decentralized wireless sensors:

1. *Energy*: the current wireless sensing designs usually adopt *ad hoc* networking that fully covers the monitoring areas with wireless sensors. This may result in the so-called data collisions. With an increasing number of sensors, the sensor node close to the sink would be burdened with tremendous data transmission levels, which may cause a significant communication and energy bottleneck.
2. *Practicability*: the fingerprint method must prepare a large training database for accurate position estimation, and the database must be frequently updated to reduce the deviation of the channel characteristics in the training and position estimation phases. This is hard in a practical underground application.
3. *Cost*: due to heavy hardware installation and network initialization calibration [15] of UWB, the cost of implementing a UWB-based WSN in a large-scale coal mine is high. In general, the length of main roadways in coal mines may range from a few to tens of kilometers [16]. For multilevel underground tunnel network structures, the total cumulative length of roadways can be hundreds of kilometers. However, the perception range of wireless sensor nodes is only tens of meters (e.g., effective perception radius of Zigbee measuring point is about 60 meters in the interior roadway environment [17]), so building a complete coverage positioning system requires high cost, which will further generate data congestion, delays, and maintenance problems due to the large number of measuring points.
4. *Reliability*: under bad underground conditions (humidity, dust, landslides, *etc.*), the personnel positioning information can be partially delayed or lost even if the area is completely covered by measuring points. Therefore, in practical applications, reliability is very demanding.

In order to effectively and efficiently manage the large number of sensors, this paper develops a new design and deployment scheme, which forms a low-cost hierarchical coal mine personnel positioning system (Mine Personnel Positioning System: MPPS) with blind areas. The main contributions of this paper are as follows:

1. A low cost coal mine personnel positioning system is designed in the tunnel environment based on Zigbee technology.
2. The data models of the underground tunnel network, the measurement network, and the moving personnel are constructed, which ground the foundation for the global positioning scheme.
3. A global positioning scheme which consists of the local positioning method, the simple correction algorithm, the three-dimensional transformation algorithm and position prediction algorithm in blind areas is proposed to obtain real-time global positioning information for the miners.

The remainder of the paper is organized as follows. In Section 2, we describe the architecture and deployment scenario of MPPS. In Section 3, the data models of global positioning systems are studied. In Section 4, we introduce the global positioning scheme. In Section 5, we analyze the prototype system and experimental results. A brief overview of related work is introduced in Section 6. Finally, we conclude the paper.

## 2. The System Architecture and Deployment Scenario

### 2.1. The System Architecture

**Figure 1.**
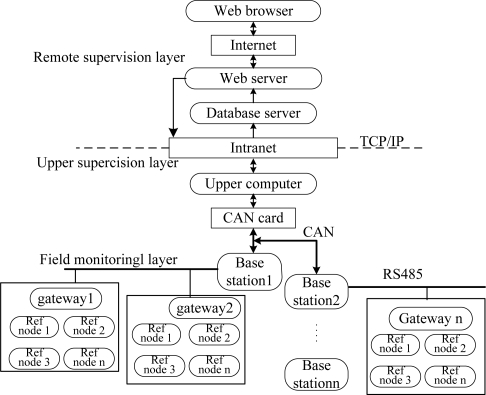
Architecture of MPPS.

As shown in Figure 1, the MPPS architecture is divided into three layers: the field monitoring layer, the upper supervision layer, and the remote supervision layer. The field monitoring layer includes the mobile nodes, the reference nodes, the gateways and the base stations. The communication between the mobile node, the reference node and the gateway is wireless. The mobile nodes continuously receive the reference nodes' location and RSSI within a one hop coverage area, calculate their own locations based on a locating algorithm, and send them to the gateway through multi-hop routing. The gateway then sends the original location information to the base station through RS-485 bus. The base station collects the location information of all miners on its own level and sends it to the upper computer after packaging through the controller area network (Controller Area Network: CAN) bus.

The upper supervision layer includes the upper computer, which is equipped with a CAN bus card in addition to a local area network (Local Area Network: LAN) card. The upper computer can communicate with the base station through the CAN card. The upper computer continuously receives the packets from the base station through the CAN interface, and then processes and visualizes these data packets. Based on the client/server model, the remote supervision level can also be divided into three layers, namely the database server layer, web server layer, and browser layer. The database server and web server are connected with the upper computer through the LAN bus complying with the TCP/IP protocol. The remote monitoring computers are connected with the web server through the Internet, and are used to monitor a variety of statistical information of MPPS through a browser.

### 2.2. The Deployment Scenario

**Figure 2.**
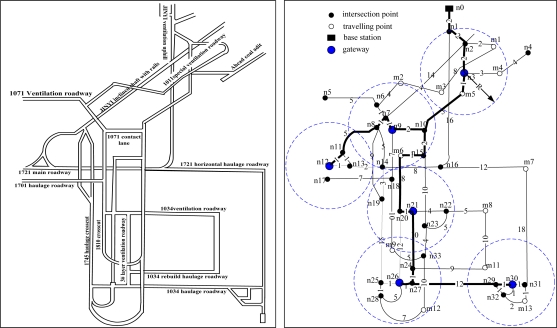
Tunnel network topology and backbone network deployment.

**Figure 3.**
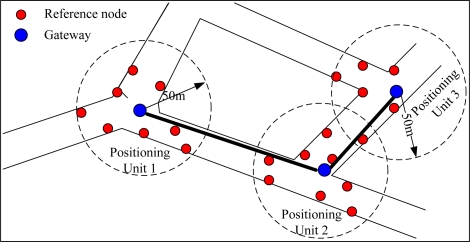
The deployment of reference nodes in each local positioning units.

The tunnel network partition for a real coal mine [18] is shown in Figure 2. To ensure that the on-site location data can be collected and processed more effectively, we divide the tunnel network into some positioning areas (denoted by dashed circles) in accordance with the distribution of the intersection and travel points. A gateway is placed in each of the local positioning area to collect and manage the mobile nodes and the reference nodes within their area, and the reference nodes are placed near the intersection points, as shown in Figure 3. The blind areas are those outside the local positioning area, where no reference nodes are placed. The wired connections between the gateways and the base station form the backbone communication network. We apply the Dijkstra algorithm and the Prim algorithm [19] to optimize the topology of the backbone network for a minimum cost. The Dijkstra algorithm is used to get the shortest path tree that connect all the gateways and base stations and the Prim algorithm is used to design a minimum spanning tree on base of the shortest path tree. The optimized result is a shortest path and minimum spanning tree as shown in Figure 2 by bold black lines.

## 3. The Data Model

To achieve the three-dimensional real-time location of personnel within the coal mine tunnel network, we have established a three-layer data model. The first layer is the tunnel network data model that creates the three-dimensional tunnel network topology; the second layer is the measurement network data model that constructs the measuring point data structure of MPPS; the third layer is the information collection data model for the establishment of the personnel location information from the mine. The data model will be achieved according to the form of data tables in the upper computer database.

### 3.1. The Tunnel Network

The tunnel network is a static spatial object, and contains all levels, intersection points, travelling points, arcs, and their topological relations in the mine. As shown in Figure 2, we define the following targets convenient for the study:

*Definition* 1: the ***Levels*** are the geo-spatial structure created by the mining in the same coal seam, which are denoted by the terms *L_evel1_, L_evel2_, ……, L_eveln_*.
*Definition* 2: the ***Arcs*** constitute the skeleton of the tunnel network, which refer to the part between every two intersections or travel points. The arcs are labelled as *A_rcID1_, A_rcID2_, ……, A_rcIDn_*.
*Definition* 3: the ***Intersection points*** are the tunnel endpoint of the arcs, indicating the intersection of tunnels at the same level, and the intersection of the tunnels and the up-down hill between different levels, which are denoted by *n_1_(x_1_,y_1_,z_1_), n_2_(x_2_,y_2_,z_2_),* ……*, n_k_(x_k_,y_k_,z_k_).*
*Definition 4*: the ***Travelling points*** are the discrete points in the arcs. There are only two arcs connected with each travelling point. The traveling points are denoted by *m_1_(x_1_’,y_1_’,z_1_’), m_2_(x_2_’,y_2_’,z_2_’),* ……*, m_k_(x_k_’,y_k_’,z_k_’).*
*Definition* 5: the ***Up-down hills*** are the roadway between neighboring levels, which are expressed by *U_pdownID1_, U_pdownID_*_2_, ……, *U_pdownIDn_*.

We set up the tunnel network topology map by calling the level table, the intersection point table, the travel point table, and the arc table, whose data structures are shown in Table 1.

In the data structure, the base types are the standard *int, long, real, string*, and *bool*. Basic conceptual entities that have been identified in spatial database research are point, line, and region [20]. In our design we defined *point, range, serial, instant* and *period* value.

**Figure 4.**
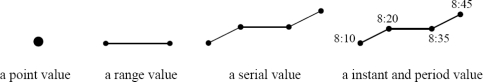
The spatial data types.

As illustrated in Figure 4, the value of type *point* represents the coordinate (*x*, *y*, *z*) of a point in the Euclidean space. The *range* value is the section between two points, which is represented by the two point values. The *serial* value is a finite set of points. The type *instant* represents a time instant. Time is considered to be linear and continuous, which has the form of (*year*, *month*, *day*, *hour*, *minute*, *second*). The type *period* is the section between two time instants, which is represented by the start instant and terminal instant.

### 3.2. The Measurement Network

The measurement network consists of the gateways, the reference nodes and the mobile nodes according to the requirement of personnel positioning. As shown in Table 2, the data structure of the gateway includes the correlative intersection point identification (CoInterPID), and the gateway's base coordinates (BaseLoc) (*i.e.*, the coordinates of the original point of each positioning unit) in the global reference frame.

In the local reference frame, the reference node data table stores its own local coordinate, and the correlative gateway identification (CoGateID). As the mobile node is carried by the miner, the mobile node ID is considered as the miner ID. The mobile node table includes the miner’s name and the configuration parameters. The ConfigPara is a user defined variable, such as the destination address, the environment parameter A and *n*, the operating mode.

### 3.3. The Personnel Positioning Information

The underground personnel positioning information includes the basic information, the real-time information and the historical information. The basic information includes the personnel identification number (MobileID), the working time in the mine (WorkTime), the working place (WorkPlace), the personnel grouping (GroupID) and other basic information. The real-time information is used to record the personnel current location, the arrival time and the current speed. The historical information consists of all underground personnel historical information saved in a central database. The variable of basic information, real-time information and historical information are shown in Table 3.

## 4. The Global Positioning Method

**Figure 5.**
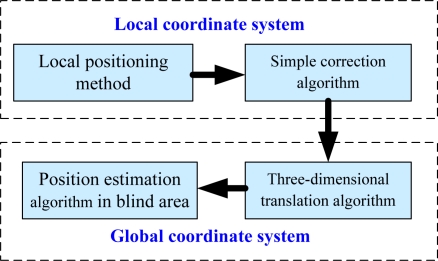
Global positioning method.

As shown in Figure 5, the global positioning method is divided into four steps: the local positioning, the simple correction, the three-dimensional translation and the position estimation in blind areas.

### 4.1. The Local Positioning Method

The field monitoring layer software estimates the real-time locations of the miners and sends them to the upper computer. The mobile node implements a distributed computation algorithm that uses RSSI values from known reference nodes. The received signal strength is a function of the transmitted power and the distance between the sender and the receiver. Here, we adopt free space radio signal attenuation model [21]:

(1) $$P_{1}/P_{2}={\left(d_{1}/d_{2}\right)}^{n}$$

where *d*_1_ and *d*_2_ are the distances between the sender and the receivers(*i.e.*, receiver 1 and receiver 2), *P*_1_ and *P*_2_ are the signal power consumption at receiver 1 and receiver 2, *n* is the path loss exponent. We can have:

(2) $$P_{1}(\mathrm{dBm})=P_{2}(\mathrm{dBm})+10n({\text{log}}_{10}(d_{1})-{\text{log}}_{10}(d_{2}))$$

Suppose *d_2_* = 1, and denote *P*_2_ (*dBm*) = *A*, *P*_1_ (*dBm*) = −*RSSI*, the Equation 2 can be expressed as:

(3) $$\mathrm{RSSI}=-(A+10n{\text{log}}_{10}\;d)$$

where *A* is the received signal strength at a distance of one meter, *d* is the distance between the sender and the receiver. The received signal power will decrease when the distance is longer. The mobile node requires the input of a set of three to eight reference coordinates with a set of measured parameters, and outputs a pair of estimated location coordinates. The local positioning method is based on the Location Engine on the chip CC2431 [22]. The local positioning procedure is as follows:

1. The mobile node broadcast one-hop RSSI requests messages in each time cycle. After receiving the messages, the reference nodes calculate RSSI values between them and reports them back to the mobile node.
2. The mobile node reads the coordinates information of related reference nodes, and starts up local positioning method with some other parameters (e.g., *A*, *n*, Collection time). It also obtains its position coordinates and sends them to the upper computer through the gateway.
3. Return to step (1), repeat the positioning process.

### 4.2. The Simple Correction Algorithm

**Figure 6.**
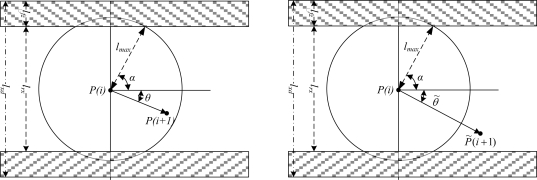
The personnel moving angle and range limitation in a mine.

Note that the maximum moving distance and direction within each sampling period is constrained by the natural conditions of the mine. As shown in Figure 6, we denote by *l_xd_* the tunnel width, *l_fz_* the width of drainage, which is called the auxiliary positioning area that is prohibited for personnel walk. The width of the pedestrian area, denoted by *l_rx_*, is named as the main positioning area.

Suppose that the position of a miner is measured by the WSN, and we denote the measured position by *P̃*(*i*) = [*x̃*(*i*), *ỹ*(*i*)]*^T^* at the sampling time *i*. Let the actual position be *P*(*i*)=[*x*(*i*),*y*(*i*)]*^T^* and the estimated position be *P̂*(*i*) = [*x̂*(*i*), *ŷ*(*i*)]*^T^*. In this paper, we define:

(4) $$i^{*}={\text{arg}}_{i}\text{ min }\{||\tilde{P}(i)-P(i)||,i=1,\cdots ,n\}$$

as the point where the measured position is closest to the actual position among *n* samplings.

In Figure 6, the left one shows the miner’s position at the next sampling time *i* + 1 should be in the circle with a radius of *l_max_*, while the right one shows the measured position *P̃*(*i*+1) at the sampling time *i* + 1. It is obvious that the measured position *P̃*(*i*+1) contains measurement errors since the miner cannot move out of the range within a sampling time or the measured position is outside of the main positioning area.

To correct the errors, we adopt the following strategies:

1. If *l_max_* is less than or equal to *l_rx_*/2, there is no intersection point between the largest moving region and the auxiliary positioning area. So we need only to judge whether *P̃*(*i*+1) is in the largest moving region or not. Outside the largest moving area, ||*P̃*(*i*+1) − *P̂*(*i*)|| is set to *l_max_* as an estimation on the miner’s position. ||*P̃*(*i*+1) − *P̂*(*i*)|| denotes the distance between the measured point at sampling time *i* + 1 and the estimate result at previous sampling time.
2. If *l_max_* is greater than *l_rx_*/2, there are intersection points between the largest moving area and the auxiliary positioning area. In this condition, we should judge whether there are intersection points between the line $\vec{\tilde{P}(i+1),\hat{P}(i)}$ and the edge of main positioning area. The measured position *P̃*(*i*+1) will be set to the intersection point *P*_0_(*i* + 1). After this, the strategy (1) will be used for correction if there is an intersection point. Based on the above correction strategy, we designed the following algorithm SimpleCorrection( ).

**Algorithm:** SimpleCorrection( )

**Input:**	$\tilde{P}{\left(i\right)}_{i=1}^{n}$, the serial measured positions;
	*l_max_* the radium of the largest moving area in a sampling period;
	*l_rx_* the width of main position area;
**Output:**	$\hat{P}{\left(i\right)}_{i=1}^{n}$ the serial estimate positions
1. work out i* = arg*_i_* min{||*P̃*(*i*) − *P*(*i*)||, *i* = 1, ⋯, *n*}	
2. for each *i* ∈ (1,2, ċ, i^*^ − 1)	
3. θ = ∠(*P̃*(i^*^ −*i*), *P̂*(i^*^ −*i*+1))	
4. *l* = ||(*P̃*(i^*^ −*i*) − *P̂*(i^*^ −*i*+1))||	
5. if *l_max_*<=*l_rx_*/2 // there is no intersection point	
6.	{if *l*<=*l_max_*
7.	Δ*x*=*l*cos(*θ*); Δ*y*=*l*sin(*θ*)
8.	else if *l*>*l_max_*
9.	Δ*x*=*l_max_*cos(*θ*); Δ*y*=*l_max_*sin(*θ*)
10.	end if
11.	*P̂*(i^*^ −*i*) = *P̂*(i^*^ −*i*+1) + [Δ*x*, Δ*y*]*^T^*
12.	}
13. if *l_max_*>*l_rx_*/2 // there is an intersection point	
14.	{if $\vec{\left(\tilde{P}(i^{*}-i),\hat{P}(i^{*}-i+1)\right)}$ and the auxiliary positioning area intersect at *P_0_*(i^*^−*i+1*)
15.	*l* = ||(*P̃*(i^*^ −*i*) − *P*_0_(i^*^ − *i*+1))||
16.	goto (line7.)
17.	else
18.	goto (line7.)
19.	}
20. for each *i* ∈ (i^*^ + 1, i^*^ + 2, ċ, *n*)	
21. θ = ∠(*P̃*(*i*), *P̂*(*i* − 1))	
22. *l* = ||(*P̃*(*i*) − *P̂*(*i* − 1))||	
23. goto (line5.)	

Note that the above algorithm SimpleCorrection( ) is used in an attempt to filter out the measurement errors in a post-processing manner because i^*^ should be worked out in advance. However if the initial position is accurately known, then i^*^=1. In such case, the algorithm is used in real time.

### 4.3. Three-Dimensional Translation Algorithm

The position obtained from the local positioning units is represented by a two-dimensional local coordinate, which is not sufficient for the global location in the tunnel network. This section will discuss how to translate the local two-dimensional coordinates into global three-dimensional coordinates.

**Figure 7.**
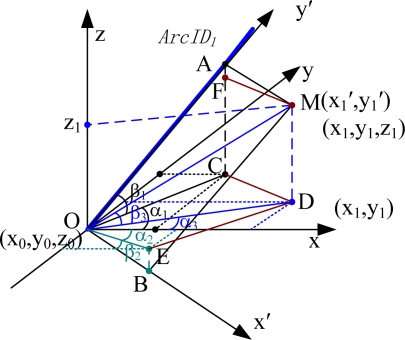
The coordinate translation method.

As shown in Figure 7, we assume that a miner (e.g., *M_obileIDi_*) is at (*x*_1_′, *y*_1_′) on *A_rcIDi_* in the local coordinate system, and send the coordinates to the upper computer through the gateway *G_ateIDj_*. The ArcAltiAng of *A_rcIDi_* in length direction (*i.e.*, the angle between *y*′ axis and its projection in the *x-y* plane) is *β*_1_, the ArcHoriAng (*i.e.*, the angle between projection of *y*′ axis in the *x-y* plane and *x* axis) is *α*_l_. The starting point of *A_rcIDi_* is the origin of the local coordinate system and the BaseLoc is (*x*_0_, *y*_0_, *z*_0_). The three-dimensional global coordinates of *M_obileIDi_* in the global coordinate system are as below:

(5) $$\{\begin{array}{l} x_{1}=x_{0}+\sqrt{\left(x_{1}^{\prime 2}+y_{1}^{\prime 2}\right)}\text{ cos }\beta_{1}\text{ cos }\alpha_{1}\\ y_{1}=y_{0}+\sqrt{\left(x_{1}^{\prime 2}+y_{1}^{\prime 2}\right)}\text{ cos }\beta_{1}\text{ sin }\alpha_{1}\\ z_{1}=z_{0}+\sqrt{\left(x_{1}^{\prime 2}+y_{1}^{\prime 2}\right)}\text{ sin }\beta_{1} \end{array}$$

To prove this, we assume that the miner’s current position is M, whose local and global coordinates are (*x*_1_′, *y*_1_′) in the *x*′-*y*′ plane, and (*x*_1_, *y*_1_, *z*_1_) in the *x*-*y*-*z* global coordinate system, respectively. D is the projection of M in the *x-y* plane, A is the projection in the *y*′ axis, B is the projection in the *x*′ axis; C is the projection of A in the *x-y* plane, E is the projection of B in the *x-y* plane.

Vector $\vec{\mathrm{OA}}$ is the length vector of the M, $\vec{\mathrm{OB}}$ is the width vector of M. The *β*_1_, *α*_1_, *β*_2_, *α*_2_ can be obtained through the tunnel network model, where *β*_1_ is the ArcAltiAng and *α*_1_ is the ArcHoriAng of *y*′ axis. and the *β*_2_, *α*_2_ are the ArcAltiAng and ArcHoriAng of *x*’ axis. Assume the ArcAltiAng and ArcHoriAng of the present personnel position vector $\vec{\mathrm{OM}}$ are *β*_3_ and *α*_3_. The *x*_1_′, *y*_1_′ have been obtained by the local positioning algorithm. According to the relationship between the vectors we can obtain the following equations:

(6) $$x_{1}=x_{0}+\bar{\mathrm{OD}}\text{ cos }\alpha_{3}=x_{0}+\bar{\mathrm{OM}}\text{ cos }\beta_{3}\text{ cos }\alpha_{3}=x_{0}+\sqrt{x_{1}^{\prime 2}+y_{1}^{\prime 2}}\text{ cos }\beta_{3}\text{ cos }\alpha_{3}$$

(7) $$y_{1}=y_{0}+\bar{\mathrm{OD}}\text{ sin }\alpha_{3}=y_{0}+\bar{\mathrm{OM}}\text{ cos }\beta_{3}\text{ sin }\alpha_{3}=y_{0}+\sqrt{x_{1}^{\prime 2}+y_{1}^{\prime 2}}\text{ cos }\beta_{3}\text{ sin }\alpha_{3}$$

(8) $$z_{1}=z_{0}+\bar{\mathrm{MD}}=z_{0}+\bar{\mathrm{OM}}\text{ sin }\beta_{3}=z_{0}+\sqrt{x_{1}^{\prime 2}+y_{1}^{\prime 2}}\text{ sin }\beta_{3}$$

(9) $$\begin{array}{l} \bar{\mathrm{CD}}=\bar{\mathrm{OE}}=x_{1}^{\prime }\text{ cos }\beta_{2}=\sqrt{{\bar{\mathrm{OC}}}^{2}+{\bar{\mathrm{OD}}}^{2}-2\bar{\mathrm{OC}}*\bar{\mathrm{OD}}\text{ cos }(\alpha_{1}-\alpha_{3})}\\ =\sqrt{{\left(y_{1}^{\prime }\text{ cos }\beta_{1}\right)}^{2}+(x_{1}{}^{2}+y_{1}{}^{2})-2y_{1}^{\prime }\text{ cos }\beta_{1}\sqrt{x_{1}{}^{2}+y_{1}{}^{2}}\text{ cos }(\alpha_{1}-\alpha_{3})} \end{array}$$

(10) $$\begin{array}{l} \bar{\mathrm{ED}}=\bar{\mathrm{OC}}=y_{1}^{\prime }\text{ cos }\beta_{1}=\sqrt{{\bar{\mathrm{OE}}}^{2}+{\bar{\mathrm{OD}}}^{2}-2\bar{\mathrm{OE}}*\bar{\mathrm{OD}}\text{ cos }(\alpha_{2}+\alpha_{3})}\\ =\sqrt{{\left(x_{1}^{\prime }\text{ cos }\beta_{2}\right)}^{2}+(x_{1}{}^{2}+y_{1}{}^{2})-2x_{1}^{\prime }\text{ cos }\beta_{2}\sqrt{x_{1}{}^{2}+y_{1}{}^{2}}\text{ cos }(\alpha_{2}+\alpha_{3})} \end{array}$$

By solving the above Equations (6∼10), the global coordinates *x*_1_, *y*_1_, *z*_1_ can be obtained. Notice that in a real coal mine, the width (3∼5 meters) of a tunnel is much smaller than the length (a few hundred meters), *i.e.*, *x*_1_′ << *y*_1_′, we can assume that *β*_3_≈*β*_1_, *α*_3_≈*α*_1_. Putting them into Equations (6∼8) then leads to the three-dimensional coordinate transformation Equation (5).

The Equation (5) is an approximate formula, which is convenient for calculating the three-dimensional coordinates when we are not able to know the ArcAltiAng (*β*_2_) and ArcHoriAng (*α*_2_) of *x*′ axis. If *α*_2_ and *β*_2_ can be precisely measured, an analytical formula is also available for the miners in underground coal mine. As shown in Figure 7, we have:

(11) $$\vec{\mathrm{OM}}=\vec{\mathrm{OA}}+\vec{\mathrm{AM}}=\vec{\mathrm{OA}}+\vec{\mathrm{OB}}$$

which can be expressed as:

(12) $$\{\begin{array}{l} x_{1}=x_{0}+x_{1}^{\prime }\text{ cos }\beta_{2}\text{ cos }\alpha_{2}+y_{1}^{\prime }\text{ cos }\beta_{1}\text{ cos }\alpha_{1}\\ y_{1}=y_{0}+x_{1}^{\prime }\text{ cos }\beta_{2}\text{ sin }\alpha_{2}+y_{1}^{\prime }\text{ cos }\beta_{1}\text{ sin }\alpha_{1}\\ z_{1}=z_{0}+x_{1}^{\prime }\text{ sin }\beta_{2}+y_{1}^{\prime }\text{ sin }\beta_{1} \end{array}$$

(13) $$\left(\begin{array}{c} x_{1}\\ y_{1}\\ z_{1} \end{array}\right)=\left(\begin{array}{c} x_{0}\\ y_{0}\\ z_{0} \end{array}\right)+\left(\begin{array}{cc} \text{cos }\beta_{2}\text{ cos }\alpha_{2} & \text{cos }\beta_{1}\text{ cos }\alpha_{1}\\ \text{cos }\beta_{2}\text{ sin }\alpha_{2} & \text{cos }\beta_{1}\text{ sin }\alpha_{1}\\ \text{sin }\beta_{2} & \text{sin }\beta_{1} \end{array}\right)\left(\begin{array}{c} x_{1}^{\prime }\\ y_{1}^{\prime } \end{array}\right)$$

Suppose that there are miners ${\left(M_{\mathrm{obileIDi}}\right)}_{i=1}^{m}$ in the arc *A_rcIDi_*, the local and global coordinates of each miner are $R_{i}={\left(x_{i}^{\prime },y_{i}^{\prime }\right)}_{i=1}^{m}$ and $G_{i}={\left(x_{i},y_{i},z_{i}\right)}_{i=1}^{m}$, respectively. *B_i_* = (*x_i_*_,0_, *y_i_*_,0_, *z_i_*_,0_) is the BaseLoc of the local coordinate system where the arc *A_rcIDi_* locates. The translation matrix *Q_i_* is:

(14) $$Q_{i}=\left(\begin{array}{cc} \text{cos }\beta_{i,2}\text{ cos }\alpha_{i,2} & \text{cos }\beta_{i,1}\text{ cos }\alpha_{i,1}\\ \text{cos }\beta_{i,2}\text{ sin }\alpha_{i,2} & \text{cos }\beta_{i,1}\text{ sin }\alpha_{i,1}\\ \text{sin }\beta_{i,2} & \text{sin }\beta_{i,1} \end{array}\right)$$

where *β_i,_*_1_ is the ArcAltiAng of *A_rcIDi_* in the length direction, *α_i,_*_1_ is the ArcHoriAng of *A_rcIDi_* in the length direction, *β_i,_*_2_ is the ArcAltiAng of *A_rcIDi_* in the width direction, *α_i,_*_2_ is the ArcHoriAng of *A_rcIDi_* in the width direction. The global coordinates of miners in *A_rcIDi_* can be easily obtained by Equation (15):

(15) $$\left(G_{1}^{T},G_{2}^{T},\cdots ,G_{m}^{T}\right)=Q_{i}\times (R_{1}^{T},R_{2}^{T},\cdots ,R_{m}^{T})+B_{i}^{T}\times (1,1,\cdots ,1)$$

### 4.4. The Position Estimation in Blind Area

As the upper computer can only get the location of miners located in the positioning areas, the positions within the blind areas need to be estimated. Suppose the personnel information from each state update is (*pid*, *t*, *arcid*, *x*, *y*, *z*, |*v*|,*θ*), where *pid* is the personnelID; *t* is the update time; *arcid* is the ArcID where *pid* located at time *t; x*, *y* and *z* are the update coordinates; |*v*| is the update velocity; *θ* is the update direction, *θ* ∈ {1, 0}, which is 1 if the direction is from the starting point to end point, (otherwise, it is 0). To simplify the explanation, we assume that personnel in the blind area moves uniformly. However, the method give in this section is also applicable for variable-speed miners (e.g., with the level of arc angle, road bumps and other factors).

#### 4.4.1. Location estimation in the past

**Figure 8.**
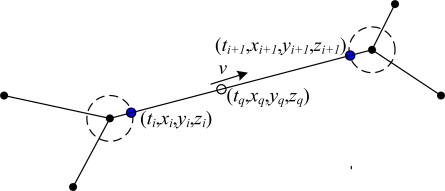
The locations are on the same arc.

As the miner is in the tunnel network, we use the linear interpolation approach to estimate the past location. As shown in Figure 8, the dashed circle represents the monitoring area of the positioning unit, outside the dashed circle is the blind area. Assume that *t_q_* is the query instant time, *t_i_* and *t_i_*_+1_ are the two adjacent update time, *arcid* is the ArcID where the miner *pid* locates at time *t_q_*, *x_q_*, *y_q_* and *z_q_* are the query coordinates. Finding the *arcid* that the miner locates at *t_q_*. If the two updates *t_i_* and *t_i_*_+1_ are both located in *arcid*, there is only one arc between two intersection nodes, the miner is located in (*t_q_*, *x_q_*, *y_q_*, *z_q_*):

(16) $$\{\begin{array}{l} x_{q}=x_{i}+((t_{q}-t_{i})(x_{i+1}-x_{i}))/(t_{i+1}-t_{i}))\\ y_{q}=y_{i}+((t_{q}-t_{i})(y_{i+1}-y_{i}))/(t_{i+1}-t_{i}))\\ z_{q}=z_{i}+((t_{q}-t_{i})(z_{i+1}-z_{i}))/(t_{i+1}-t_{i})) \end{array}$$

**Figure 9.**
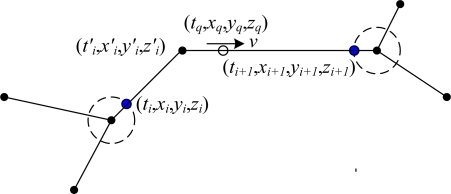
The locations are on the different arcs.

If the two updates are not in the same arc, (see Figure 9), we can calculate the distance from *t_i_* to *t_q_* as *L* = |*v*| * (*t_q_*−*t_i_*), where *t_i_* is the previous update time and *v* is the rate. According to the intersection node model, the number of arcs, the ordered sequence, and the length of each arc have been defined, therefore, the A_rcIDi_ at the time *t_q_* is determined. Then the state information is determined as (*t_i_*′, *x_i_*′, *y_i_*′, *z_i_*′) and (*t_i+1_*, *x_i+1_*, *y_i+1_*, *z_i+1_*), respectively, when the miner reached the travel points at both terminals of A_rcIDi_. Finally, the following formula will be used to determine the location at time *t_q_*:

(17) $$\{\begin{array}{c} x_{q}=x_{i}+((t_{q}-t_{i}^{\prime })(x_{i+1}-x_{i}^{\prime }))/(t_{i+1}-t_{i}^{\prime }))\\ y_{q}=y_{i}+((t_{q}-t_{i}^{\prime })(y_{i+1}-y_{i}^{\prime }))/(t_{i+1}-t_{i}^{\prime }))\\ z_{q}=z_{i}+((t_{q}-t_{i}^{\prime })(z_{i+1}-z_{i}^{\prime }))/(t_{i+1}-t_{i}^{\prime })) \end{array}$$

#### 4.4.2. Estimation of present and future positions

To estimate the present and future positions based on the previous movement, we can use several forecasting models:

1. Delay model:

(18) $${\hat{v}}_{n}=v_{n-1}$$

The rate value and direction of the next time are the same as the previous ones.

2. Moving average model:

(19) |v^n|=∑i=1m|vn−i|mθn=θn−1

The scalar value of the speed of the next time is the mean value of the previous *m* ones, its direction is the same as the last historical one *v*_n−1_. Obviously, the delay model is more sensitive to the changes in personnel movement status, while the moving average model is more accurate for the scalar speed prediction.

According to the above two speed models, we can predict the position at the time *t_q_*∈(*t*_n_, *t*_n +1_). It is also needed to estimate the arcs based on the historical position logs, and then estimate the position based on the previous position, the speed and direction information. According to the above estimation strategy, we designed the following algorithm PositionEstimation( ).

**Algorithm 2.** PositionEstimation( )

**Input:**	*mgpoint*= ${\left(pid_{i},t_{i},\;\mathrm{arci}d_{i},\;x_{i},\;y_{i},\;z_{i},\left|v_{i}\right|,\theta_{i}\right)}_{i=1}^{n}$, the update log in database;
	*G* the tunnel network graph;
	*t_q_* the query instant time;
	*pid* the personnel ID who be queried;
**Output:***gp_q_*={*arcid_q_, x_q_, y_q_, z_q_*} the location of *pid* at *t_q_*;	
1. for each update log ${\left(pid_{i},t_{i},\;\mathrm{arci}d_{i},\;x_{i},\;y_{i},\;z_{i},\left|v_{i}\right|,\theta_{i}\right)}_{i=1}^{n}$;	
2. if ∃ *i* ∈ {1, 2, …, *n*}: *t_q_= t_i_* ;	
3. output *gp_q_*={*arcid_i_*, *x_i_*, *y_i_*, *z_i_*}	
4. else if ∃ *i* ∈ {1, 2, …, *n*-1}: *t_i_< t_q_< t_i+_*_1_ ;	
5.	{if *t_i_* and *t_i_*_+1_are located in the same arc
6.	Output *arcid_q_= arcid_i_*,
	$$\{\begin{array}{c} x_{q}=x_{i}+((t_{q}-t_{i})(x_{i+1}-x_{i}))/(t_{i+1}-t_{i}))\\ y_{q}=y_{i}+((t_{q}-t_{i})(y_{i+1}-y_{i}))/(t_{i+1}-t_{i}))\\ z_{q}=z_{i}+((t_{q}-t_{i})(z_{i+1}-z_{i}))/(t_{i+1}-t_{i})) \end{array}$$
7.	else // *t_i_* and *t_i_*_+1_ are not located in the same arc
8.	{determine the *arcid_i_* in which *pid* located at *t_q_*;
9.	determine the state (*t*_j_, *x*_j_, *y*_j_, *z*_j_) and (*t*_j+1_, *x*_j+1_, *y*_j+1_,*z*_j+1_) at each end of *arcid_i_*;
10.	put the state in step (9) into the Equation 16 to estimate the location;
11.	}
12.	}
13. else // *t_n_< t_q_*	
14.	{ $L=\left|v\right|*(t_{q}-t_{n})=\left(\sum_{i=1}^{m}\left|v_{n-i}\right|\right)*(t_{q}-t_{n})/m$;
15.	Predict the *arcid_i_* in which *pid* located at *t_q_*;
16.	Predict the state (*t*_j_, *x*_j_, *y*_j_, *z*_j_) and (*t*_j+1_, *x*_j+1_, *y*_j+1_, *z*_j+1_) at each end of *arcid_i_*;
17.	put the state in step (16) into the linear interpolation Equation 16 to predict the location;
18	}
19. end	

## 5. Experimental Results

### 5.1. MPPS Prototype

To verify the feasibility of the proposed global positioning system with blind areas, we have independently developed a prototype MPPS system. The hardware and the embedded software (firmware) of the base stations, the gateways, the reference nodes, and the mobile nodes were independently developed with the IAR Embedded Workbench IDE 7.30. The upper supervision software and remote supervision software are developed with Microsoft Visual C++ 6.0.

#### 5.1.1. The Gateway

**Figure 10.**
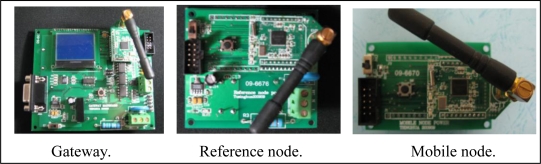
The devices in the positioning unit.

The gateway is the center of the positioning unit. As shown in Figure 10, the hardware of the gateway includes a kernel board that integrates a microprocessor and an antenna, and a motherboard containing a liquid crystal display (Liquid Crystal Display: LCD), the power and the communication interfaces. The microprocessor (CC2430 [23]) receives and transmits the personnel positioning and configuration information, ensures that the task is performed at the correct interval, packages the raw data into a message, and sends the message to the radio hardware. The microprocessor provides extensive hardware support for packet handling, data buffering, concurrent transmission, data encryption, data authentication, clear channel assessment, link quality indication and so on.

#### 5.1.2. The Reference Node

The reference node is placed within the wireless signal coverage area of the gateway to provide a package with its own X, Y coordinates and RSSI values to mobile nodes, so it should be configured with high accuracy. The reference node hardware, as shown in Figure 10, contains a power board and a kernel board.

#### 5.1.3. The Mobile Node

The mobile node can communicate with the nearby reference nodes, collect these nodes' X, Y coordinates and RSSI values, calculate its own location based on these data and input parameters A, n, and then send the location to the gateway. As shown in Figure 10, the mobile node uses the positioning chip CC2431 [22] to execute the above mission.

#### 5.1.4. The Upper Supervision

The upper supervision software consists of the data collection module, the information extraction module, the data storage module and the data display module. The data collection module receives the information packets from the base station and stores them into the different message queue pools. The data display module is used to generate the topology of the coal mine tunnel network and visualize the personnel and node location information. By inputting the parameters of intersection points, travel points and arcs into the software dialog box according to the actual geographical tunnel structure, the tunnel topology diagram will be generated automatically.

#### 5.1.5. The Remote Supervision Software

**Figure 11.**
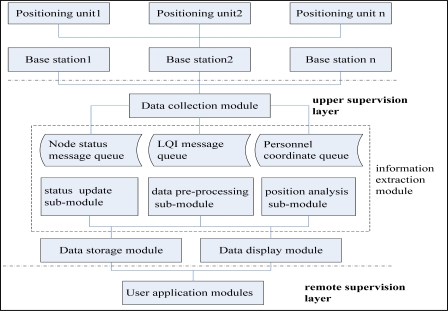
Software structure of the MPPS.

The main task of the remote supervision platform is to display the current network status in real-time, including the status of the gateways, the reference nodes and the mobile nodes, and the location information of the mobile miners. The client users can learn the network information and retrieve the current and historical underground personnel information by searching under a keyword via the remote supervision platform. The software structure of MPPS is shown in Figure 11.

### 5.2. Static Positioning Experiment

**Figure 12.**
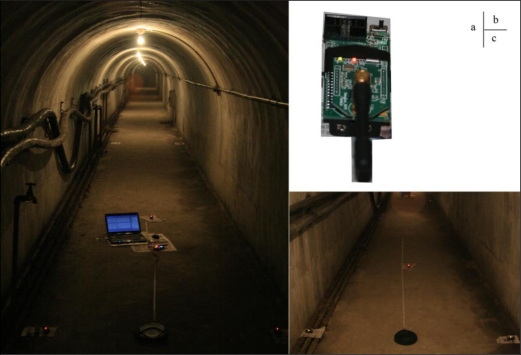
The deployment of positioning system in a underground tunnel.

As shown in Figure 12(a), a static positioning experiment was carried out in an underground tunnel. The experimental system consists of a positioning unit (*i.e.*, a gateway, four reference nodes and a mobile node), and an upper computer (*i.e.*, a notebook PC: Windows 7 operating system, Intel Core 2 Duo 1.8 GHz processor, 2 GB memory, 320 GB hard disk). The positioning unit was installed in the tunnel, and is connected to the upper computer through RS232 cable. Figure 12(b) is a reference node in operation. We deploy the measurement nodes as shown in Figure 12(c).

**Figure 13.**
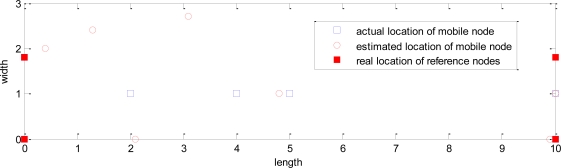
Static positioning performance, *A* = 40, *n* = 3.875.

The average temperature in the tunnel is 13 °C, the average relative humidity is above 100%, the width is 1.8 meters, the length is about 160 meters, the vertical angle is about 0°, and the horizontal angle is about 30°, which is close to the actual environment of underground mines. As shown in Figure 13, the length direction and width direction are set as the x’-axis and y’-axis, respectively, and the tunnel entrance as the coordinate origin to establish a local reference frame. Four reference nodes were arranged along the tunnel edges, whose coordinates are R1 (0, 0), R2 (0, 1,75), R3 (10, 0), R4 (10, 1.75), and record the coordinates of the mobile node. The location engine implemented in CC2431 is not using *n* directly, instead it is using a value termed n_index. The relation between *n* and n_index can be seen in the Table 4 [22].

The empirical parameter *A* is set to 40, *n* is set to 3.875, and the test results are shown in Table 5 (To obtain more objective results, we continuously sampled 10 sets of data at each location, and calculate the average).

The test error in Table 4 is defined as:

(20) $$\text{Err(i)}=\sqrt{{\left(x_{\mathrm{real}}\;(i)-x_{\mathrm{est}}\;(i)\right)}^{2}+{\left(y_{\mathrm{real}}\;(i)-y_{\mathrm{est}}\;(i)\right)}^{2}}$$

The average error is defined as:

(21) E¯=∑i=1mErr(i)m

where the (*x_real_*, *y_real_*) is the actual position, the (*x_est_*, *y_est_*) is the test result, *m* is the sample number. The three-dimensional global positions can also be determined according to the coordinate transformation (5) or (13). The maximum test error of the experiment positioning system was 2.1 meters at the position (0.0, 0.0). The average error *E̅* = (2.1 + 1.3 + 1.9 + 1.9 + 0.2 + 0.1)/7 = 1.1 meters, which meets the common design tolerance of 3∼5 meters positioning error in underground personnel positioning situations.

However, the RSSI-based location estimation in 802.15.4 may be drastically affected by the parameter *A* and *n* in Equation (3). The parameter named *n* is a parameter that describe how the signal strength decreases when the distance from the transmitter increases. *n* is highly dependent of the environment [24]. We did two experiments to testify the effect of the parameter *A* and *n*, respectively. First, we set *n* to 3.875, and change *A* from 30 to 45. The measurement results and test errors are shown in Table 6. It can be seen that the estimation errors vary with *A*. Generally, an *A* value between 40 and 45 gives the most accurate answer for the tunnel environment.

In the other experiment, we set *A* to 40 and change *n* from 3.375 to 4.625. The experimental results show that the positioning errors varies with the *n* in Table 7. Generally, an *n* value between 3.875 and 4.125 gives most accurate results.

### 5.3. The Global Positioning Experiment of MPPS

**Figure 14.**
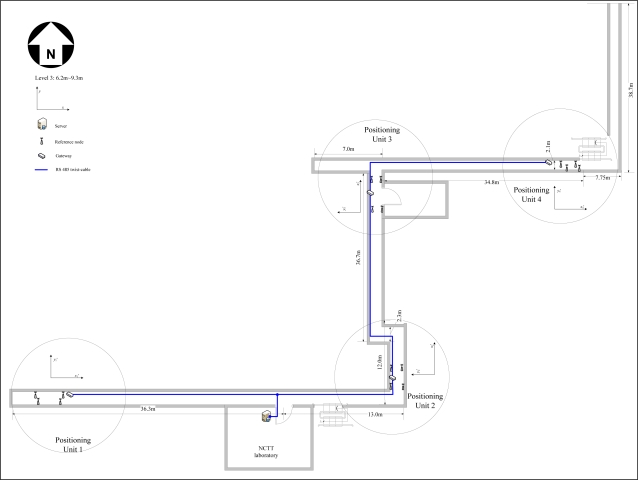
The deployment of positioning system in a corridor.

In order to verify the overall performance of MPPS, we installed twelve positioning units in the corridor of the main building in Tsinghua University. Figure 14 is the deployment map of network devices in the third level (3rd floor). The upper computer (*i.e.*, the server) was located in NCTT laboratory, and each monitoring area (circle as shown in Figure 14) was placed a gateway and four reference nodes. The gateways and server were connected via RS-485 twist-cables, the reference nodes and the gateway were wirelessly connected according to the Zigbee protocol. The reference nodes were configured as shown in Table 8. The configuration parameters of the mobile node are listed in Table 9. Positioning units in level 1, 2 were deployed as the same as level 3.

In Table 8, we set the length direction as the x’-axis, the width direction as the y’-axis of each local positioning unit. The coordinates of reference nodes denote the coordinates in the local reference frame (e.g., ${\left({x^{\prime }}_{i}-{y^{\prime }}_{i}\right)}_{i=1}^{4}$ in Figure 14). The Refnode 1 is set as the coordinate origin of the local coordinate system, the Refnode 2∼4 are the other three reference nodes in the counterclockwise direction.

The configure parameters are chosen as shown in Table 9, where the empirical parameter *A*, can be determined by measuring the RSSI value one meter from the transmitting unit. The operation mode is set to 0, which denotes the mobile node only sends responses to requests; if it is set to 1, it automatically sends REFNODE responses according to the Cycle time (in seconds). The collection time is the time used on waiting for reference node response, which is in 100 millisecond increments. Min. Ref. Nodes is the minimum number of reference nodes that are needed for local positioning algorithm. We chose these parameters according to Table 9.

The following steps are used to validate the performance of MPPS:

1. Configure the gateways, the reference nodes and the mobile nodes in the upper computer remotely.
2. Collect the personnel coordinate information in the corridors.
3. Display the real-time location information of the mobile nodes.
4. Query the location information of special personnel in the future instant or the historical period.

**Figure 15.**
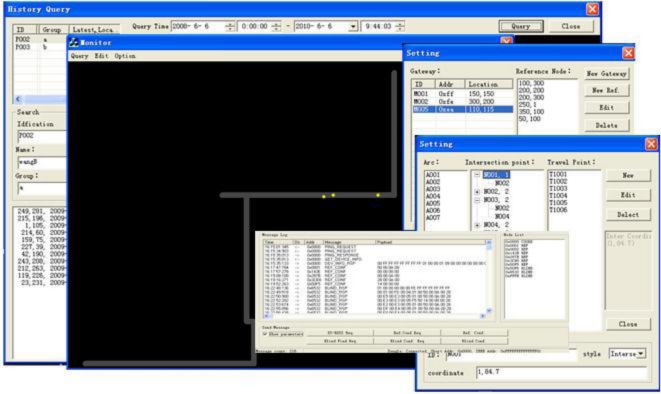
A snapshot of the tunnel network, the message log and parameter configuration.

A snapshot of the tunnel network, the message log and parameter configuration on the upper computer is shown in Figure 15. The experimental results show that the hardware and software of MPPS work properly, the location information of mobile nodes was translated from the gateway to the upper computer in real-time, and periodically refreshed on the display screen (refresh rate 2 seconds). The reference nodes and mobile nodes can also be remotely configured on the upper computer; the personnel positioning information can be correctly translated among the positioning units, the upper computer and the remote client computer. The historical path of specific personnel can be shown in the form of the data tables or curves. The test error within the coverage area is approximately 3 meters. For personnel moving along the corridor with uniform walking speed, the estimation error is less than 4.5 meters according to the delay model, which meets the basic positioning requirements.

### 5.4. The Comparison between MPPS, RFID and Zigbee Positioning System

Next, we compare MPPS with an existing RFID positioning system (e.g., KJ280: personnel positioning system in coal mine [25]) and a Zigbee positioning system (e.g., KJ272: personnel positioning system in coal mine [26]). As shown in Table 10, MPPS has some advantages in the function, the positioning precision, the cost, the reliability and maintainability.

## 6. Related Work

Localization has been studied for many years as a classical problem in many disciplines, including navigation systems (VOR [27]) and GPS [28]), user location identifying in cellular networks [29,30] and WLANs [31–33], and the robot localization in mobile robotics [34,35]. However, solutions for the above problems may not be directly applicable to underground coal mine sensor networks. In this section, we provide a brief literature review. First, we introduce some classical positioning methods [36], including TOA [37], Time Difference of Arrival (TDOA) [38] Angle of Arrival (AOA) [39], and RSSI [33]. Then, we consider in particular the blind spot prediction systems.

### 6.1. The Positioning Methods

For localization systems, GPS is a good solution in outdoor environments. However, installing a GPS receiver on each sensor node may not be a practical solution for most applications because of the size, the battery, the cost, and the environment constraints of sensor nodes. The “GPS-free” localization system has attracted significant research effort in recent years and many approaches have been proposed.

RF TOA is a common technology used to measure distance via signal propagation time. The most basic localization system using TOA is the GPS, which requires relatively expensive and energy-consuming electronics to precisely synchronize with a satellite’s clock [40]. But the results may be highly biased by the timing inaccuracy caused by any processing delays [41], multipath and nonline of sight (NLOS).

TDOA uses the difference between the traveling times of two signals to estimate distance. For example, in Cricket [38], Nissanka *et al.* measure the time difference between two simultaneously transmitted radio and ultrasound at the receiver. Generally, TDOA gives more accurate distance estimations than TOA since the second medium (for example, ultrasound) travels at a much slower speed, making it not as sensitive to timing as TOA. However, equipping ultrasound to a sensor node not only means more cost and energy consumption but also requires nodes to be densely deployed (ultrasound usually only propagates to 20–30 feet), which makes it unsuitable for coal mine applications.

In AOA measurement, directive antennas or antenna arrays are used to estimate the angle of arrival of the received signal from a beacon node. The concept of AOA was originally used in the VOR/VORTAC system for aircraft navigation. When used for sensor positioning, two factors have to be considered: (1) AOA can be difficult to measure accurately if a sensor is surrounded by scattering objects and (2) the required directive antennas or antenna arrays for AOA measurement may become prohibitive for tiny or cheap sensor nodes.

RSS has been widely used as a distance measure in the context of WSNs because of its attractive features. It does not require any additional hardware since the RSSI is a standard feature of the communication system, thus reducing sensor size, cost and not significantly impacting on the local power consumption of the device [42]. In the RSS method, the measured received power and the known transmitted power are used to determine the channel path loss, which is highly correlated with the path length. RSS-based localization has been studied extensively [43–45]. Whitehouse and Culler [46,47] designed and evaluated an *ad hoc* localization system called Calamari which provided an important observation about RSS measurements: although it is well known that RSS is unreliable in complex indoor or urban environments, many sensor network applications are situated in ideal settings for measuring RSS. Furthermore, calibration can be used to highly enhance the accuracy of RSS measurements. In this paper, we adopt the RSSI-based location engine as the fundamental factor in global positioning method.

### 6.2. Blind Spot Prediction System

Most of today’s land vehicles are equipped with GPS to provide accurate position and velocity information. However, GPS has limitations such as low sampling rate and it is difficult to obtain continuous localization since the satellite signal may be lost and corrupted due to high buildings, tunnels and mountains, multi-path reflections and bad weather conditions [48]. Therefore, GPS is usually combined with Inertial Navigation System (Inertial Navigation System: INS), which is a self-contained system that calculates the position, velocity, and attitude of a vehicle with the output of inertial sensors. The integration of GPS and INS, therefore, provides a navigation system that has superior performance in comparison with either a GPS or an INS stand-alone system. For instance, GPS position components have approximately white noise characteristics with bounded errors and can therefore be used to update INS and improve its long-term accuracy. On the other hand, INS provides positioning information during GPS outages thus assisting GPS signal reacquisition after an outage and reducing the search domain required for detecting and correcting GPS cycle slips.

The Kalman filter (KF) has been widely adopted as the optimal estimation tool of the GPS/INS integration scheme for many land vehicle navigation and positioning applications [49–52]. The major inadequacy related to the utilization of KF for GPS/INS integration is the necessity to have a predefined accurate stochastic model for each of the sensor errors and prior information about the covariance values of both INS and GPS data accurately. More recently, several techniques based on Artificial Intelligence (AI) have been proposed to replace KF in order to eliminate some of its inadequacies [53–56]. The AI-based method is to mimic the latest vehicle dynamics by training the AI module during the availability of the GPS signals. In case of GPS outages, an empirical model that processes the INS output and provides the corresponding INS position operates in the prediction mode to correct for inaccuracies in INS outputs.

Moreover, an INS/GPS/SAR integrated navigation system proposed in [57], which represents the trend of next generation navigation systems, offers high independence, performance, high precision and reliability. Synthetic Aperture Radar (SAR) is a new sensing technique using active microwave imaging radar, which overcomes the limitations of GPS, and the obtained high precision images can be used to correct the errors of INS according to the identified target information [58]. In [59], a GPS, Zigbee, and the Google-Earth engine-based Assisted Driving System (ADS) is proposed for haul trucks operating in surface mining and construction sites. To improve the positioning accuracy, DGPS [60] and WADGPS [61] are also designed. However, equipping GPS receivers or INS for underground miners is expensive and not convenient, a GPS-free positioning scheme is greatly demanding.

## 7. Conclusions

A low-cost coal mine personnel global positioning system with blind areas (MPPS) based on wireless sensor networks (WSN) has been presented. The measurement of the miners’ location only requires sensors placed in the tunnels’ key spots, while their global positions are numerically estimated in the underground tunnel network. This greatly reduces the cost of the system. Several key technical issues are addressed by this proposed system such as positioning unit hardware, upper computer and remote supervision software, data model, and global positioning method.

A prototype of MPPS was designed, and the experimental results of both the static and dynamic positioning show that the prototype hardware and software can work properly with a static positioning error below 2.6 meters and adynamic estimation error below 4.5 meters, which meet the preliminary design requirements. Further efforts are necessary to improve the QoS of wireless communication, reliability of measuring nodes, and standardization of interfaces and interoperability. In addition, further studies of positioning algorithm of different conditions in underground coal mine are necessary to determine the specific limitations and possible new applications of this technology.

## Figures and Tables

**Table 1.** The level, the intersection point, the travel point and the arc data structure.

**Level**	**Intersection point**	**Travel point**	**Arc**
LevelID: int	NodeID: int	TravID: int	ArcID: int
LevHorRan: *range*	Location: *point*	Location: *point*	StartNodeID: int
LevVerRan: *range*	ArcNum: int	BackTravPID: int	EndNodeID: int
NodeNum: int	ArcIDs: *serial*	AheadTravPID: int	ArcWide: real
NodeIDs: *serial*	NabNodNum: int	ArcID: *serial*	ArcLength: real
	NabNodIDs: *serial*		ArcAltiAng: real
			ArcHoriAng: real

**Table 2.** Gateway, reference node and mobile node data structure.

**Gateway**	**Reference node**	**Mobile node**
GateID: int	RefID: int	MobileID: int
GateAddr: long	RefAddr: long	MobileAddr: long
CoInterPID: int	CoGateID: int	Name: string
BaseLoc: *point*	LocalLoc: *point*	ConfigPara: *user defined*

**Table 3.** The personnel information data format.

**Basic information**	**Real-time information**	**Historical information**
MobileID: int	MobileID: int	MobileID: int
Name: string	CurrentLoc: *point*	GateID: int
GroupID: int	CurrentTime: *instant*	InTime: *instant*
WorkPlace: string	CurrentVelo: real	OutTime: *instant*
WorkTime: *period*	CurrentVeloDir: bool	InArcID: int
		OutArc: int
		AverVelo: real

**Table 4.** *n* parameter lookup table.

n_index	1	2	3	4	5	6	7	8	9	10	11	12	13
*n*	1.250	1.500	1.750	1.875	2.000	2.125	2.250	2.375	2.500	2.625	2.750	2.875	3.000
n_index	14	15	16	17	18	19	20	21	22	23	24	25	26
*n*	3.125	3.250	3.375	3.500	3.625	3.750	3.875	4.000	4.125	4.250	4.375	4.500	4.625

**Table 5.** The experiment test results of positioning system (in meters).

	**Location 1**	**Location 2**	**Location 3**	**Location 4**	**Location 5**	**Location 6**	**Location 7**
Actual position	(0.0, 0.0)	(0.0, 1.8)	(2.0, 1.0)	(4.0, 1.0)	(5.0, 1.0)	(10.0, 0.0)	(10.0,1.0)
Test result	(2.1, 0.0)	(0.4, 2.0)	(1.3, 2.4)	(3.1, 2.7)	(4.8, 1.0)	(9.9, 0.0)	(10.0,1.0)
Test error	2.1	1.3	1.9	1.9	0.2	0.1	0.0

**Table 6.** The experiment test results and error (in meters), *n* = 3.875.

	**Location 1**	**Location 2**	**Location 3**	**Location 4**	**Location 5**
Actual position	(0.2, 1.0)	(3.0, 1.0)	(5.0, 1.0)	(7.0, 1.0)	(9.0, 1.0)
Test result and error (*A*=30)	(1.8, 5.5), 4.8	(0.3, 8.8), 8.3	(5.2, 8.1), 7.1	(3.1, 8.9), 8.8	(13.8, 4.6), 6.0
Test result and error (*A*=35)	(3.4, 0.4), 3.2	(1.6, 6.6), 5.7	(5.7, 4.2), 3.3	(4.3, 6.7), 6.8	(12.8,3.1), 4.3
Test result and error (*A*=40)	(2.3, 0.6), 2.1	(3.7, 2.9), 2.1	(5.5, 1.1), 0.5	(5.0, 2.4), 2.4	(8.4, 0.6), 0.7
Test result and error (*A*=45)	(2.5, 0.1), 2.4	(3.5, 0.0), 1.1	(8.6, 4.3), 4.9	(5.0, 0.0), 2.2	(7.6, 0.4), 1.5

**Table 7.** The experiment test results and error (meter), *A* = 40.

	**Location 1**	**Location 2**	**Location 3**	**Location 4**
Actual position	(3.0, 1.0)	(5.0, 1.0)	(7.0, 1.0)	(9.0, 1.0)
Test result and error (*n* =3.375)	(0.3, 4.8), 4.4	(7.4, 4.9), 4.6	(4.4, 6.2), 5.8	(11.0, 3.7), 3.4
Test result and error (*n* =3.625)	(0.0, 4.4), 4.5	(6.8, 5.8), 5.1	(4.4, 3.1), 3.3	(9.3, 4.0), 3.0
Test result and error (*n* =3.875)	(3.7, 2.9), 2.1	(5.5, 1.1), 0.5	(5.0, 2.4), 2.4	(8.4, 0.6), 0.7
Test result and error (*n* =4.125)	(2.7, 0.0), 1.1	(6.0, 1.3), 1.0	(5.0, 0.4), 2.1	(7.3, 2.5), 2.3
Test result and error (*n* =4.375)	(3.0, 0.0), 1.0	(6.0, 1.0), 1.0	(5.0, 0.0), 2.2	Overrun
Test result and error (*n* =4.625)	(3.0, 0.1), 0.9	(6.8, 1.6), 1.9	(5.0, 0.0), 2.2	Overrun

**Table 8.** The configuration parameters of the reference nodes in the third level.

	L_evelID_	BaseLoc	Refenode 1	Refenode 2	Refenode 3	Refenode 4
**Positioning Unit 1**	3	(4.0, 6.0)	(0.0, 0.0)	(4.0, 0.4)	(4.2, 2.0)	(0.2, 1.9)
**Positioning Unit 2**	3	(49.3, 9.0)	(0.0, 0.0)	(3.2, 0.6)	(3.0, 1.9)	(0.5, 2.0)
**Positioning Unit 3**	3	(47.2, 51.7)	(0.0, 0.0)	(3.8, 0.3)	(3.6, 1.8)	(0.3, 2.2)
**Positioning Unit 4**	3	(78.1, 54.7)	(0.0, 0.0)	(3.6, 0.2)	(3.8, 2.0)	(0.2, 2.0)

**Table 9.** The configuration parameters of the mobile nodes.

	A	*n*	Operation mode	Collection time	Cycle time	Min. Ref. Nodes
**Mobile node**	39	3.875	0	10	2	3

**Table 10.** The comparison between KJ280, KJ272 and MPPS positioning system.

**System**	KJ280	KJ272	MPPS
**Architecture**	Fieldbus architecture	Clustering structure	Clustering-Fieldbus hybrid hierarchical architecture with blind areas
**Function**	Location registration, Location query	Location registration, Location query	Location registration, Integrated information query, Location prediction
**Positioning method**	Radio Frequency Identification Technology	Received signal strength indication–based location method	Four-step global positioning method: the local positioning, the simple filtering, the three-dimensional algorithm and the estimation algorithm in blind area
**Precision**	Regional Location	≤ ±15 meters	≤ ±3.0 meters (positioning areas); ≤ ±4.5 meters (blind areas)
**Cost**	High	High	Low
**Reliability**	Medium	Low	High
**Power**	Mains supply	Batteries	Mains supply and batteries
**Maintainability**	Senior RFID reader is complex and expensive, complex maintenance	Arrange a large number of routing nodes to maintain the wireless connectivity, complex maintenance	Measuring nodes decreased greatly due to the blind areas, easy maintenance
